# Clusterin Is a Potential Lymphotoxin Beta Receptor Target That Is Upregulated and Accumulates in Germinal Centers of Mouse Spleen during Immune Response

**DOI:** 10.1371/journal.pone.0098349

**Published:** 2014-05-27

**Authors:** Marina A. Afanasyeva, Liudmila V. Britanova, Kirill V. Korneev, Nikita A. Mitkin, Anna A. Kuchmiy, Dmitry V. Kuprash

**Affiliations:** 1 Engelhardt Institute of Molecular Biology, Russian Academy of Sciences, Moscow, Russia; 2 Department of Immunology, Faculty of Biology, Lomonosov Moscow State University, Moscow, Russia; McGill University, Canada

## Abstract

Clusterin is a multifunctional protein that participates in tissue remodeling, apoptosis, lipid transport, complement-mediated cell lysis and serves as an extracellular chaperone. The role of clusterin in cancer and neurodegeneration has been extensively studied, however little is known about its functions in the immune system. Using expression profiling we found that clusterin mRNA is considerably down-regulated in mouse spleen stroma upon knock-out of lymphotoxin β receptor which plays pivotal role in secondary lymphoid organ development, maintenance and function. Using immunohistochemistry and western blot we studied clusterin protein level and distribution in mouse spleen and mesenteric lymph nodes in steady state and upon immunization with sheep red blood cells. We showed that clusterin protein, represented mainly by the secreted heterodimeric form, is present in all stromal compartments of secondary lymphoid organs except for marginal reticular cells. Clusterin protein level rose after immunization and accumulated in light zones of germinal centers in spleen - the effect that was not observed in lymph nodes. Regulation of clusterin expression by the lymphotoxin beta signaling pathway and its protein dynamics during immune response suggest a specific role of this enigmatic protein in the immune system that needs further study.

## Introduction

Lymphotoxin beta receptor (LTβR) signaling plays a crucial role in development of secondary lymphoid organs (SLO). Surface lymphotoxin (LT) is a transmembrane heterotrimeric protein that belongs to the tumor necrosis factor (TNF) family and is expressed by lymphoid tissue inducer cells during early phases of SLO formation [1]. Acting through LTβR on lymphoid tissue organizer cells and earlier on their mesenchimal precursors, it activates synthesis of chemokines, adhesion molecules and lymphangiogenic factors through classical and alternative NFκB pathways, leading to maturation of stroma and lymphocyte homing [2], [3], [4]. In postnatal period, LTβR signaling is required for follicular dendritic cell (FDC) maintenance and germinal center (GC) formation in lymph nodes [5]. And it is even more important in spleen, where postnatal LTβR-Ig treatment leads to disruption of follicles and marginal zone, as well as GC failure [6].

Clusterin (also known as testosterone repressed prostate message-2, sulfated glycoprotein-2, apolipoprotein J (ApoJ), and X-ray–inducible transcript 8) was first described as the major glycoprotein in ram rete testis fluid with the capacity to elicit clustering of cells in an *in vitro* assay [7]. It is a multifunctional protein, which is mainly studied for its role in neurodegeneration and cancer [8]. Its mRNA is present at relatively high levels in brain, ovary, testes, liver, heart and adrenal gland; at moderate levels in spleen, lung, breast, kidney, seminal vesicle, prostate, and uterus; at low levels in skin, bone, thymus and digestive tract; and is absent in T-lymphocytes [9], [10]. Clusterin participates in tissue remodeling, apoptosis, lipid transport, complement-mediated cell lysis, and serves as an extracellular chaperone [8], [11], [12], [13], [14], [15], [16].

At the protein level, clusterin was found in non-lymphoid cells of many SLO: gut-associated lymphoid tissue, Waldeyer's ring [17], [18], reactive tonsils, lymph nodes and spleen [5], [19], but virtually nothing is known about its function in these organs. Clusterin is also present in medullary epithelial stromal cells of the primary lymphoid organ - thymus, but its precise function there is also not clear [20], [21].

In the present work we used expression profiling to identify new potential target genes of LTβR signaling pathway by comparing transcriptomes of spleen stromal cells derived from wild type and LTβR knock-out (LTβR-KO) mice. Since LTβR signaling drives morphogenesis and functional maturation of SLO, we expected to find new immunity-relevant genes among its targets. After filtration of the microarray results we focused on clusterin as it was significantly downregulated in LTβR-deficient spleen at both mRNA and protein level and its function in the immune system was poorly studied. We demonstrated activation of clusterin gene transcription upon interaction of mouse embryonic fibroblasts (MEF) with lymphoid cells bearing LT and significant changes in clusterin protein level and tissue distribution during primary immune response to T-dependent antigen.

## Results and Discussion

### Transcriptome of splenic stromal cells in normal and LTβR-deficient mice

LTβR-KO mice lack all lymph nodes and Payer's patches. Their spleen is completely disorganized with no proper segregation into red and white pulp. Nevertheless, lymphocytes are present in the organ, but they are not organized in conventional T- and B-zones. GCs also fail to develop [22].

**Figure 1:**
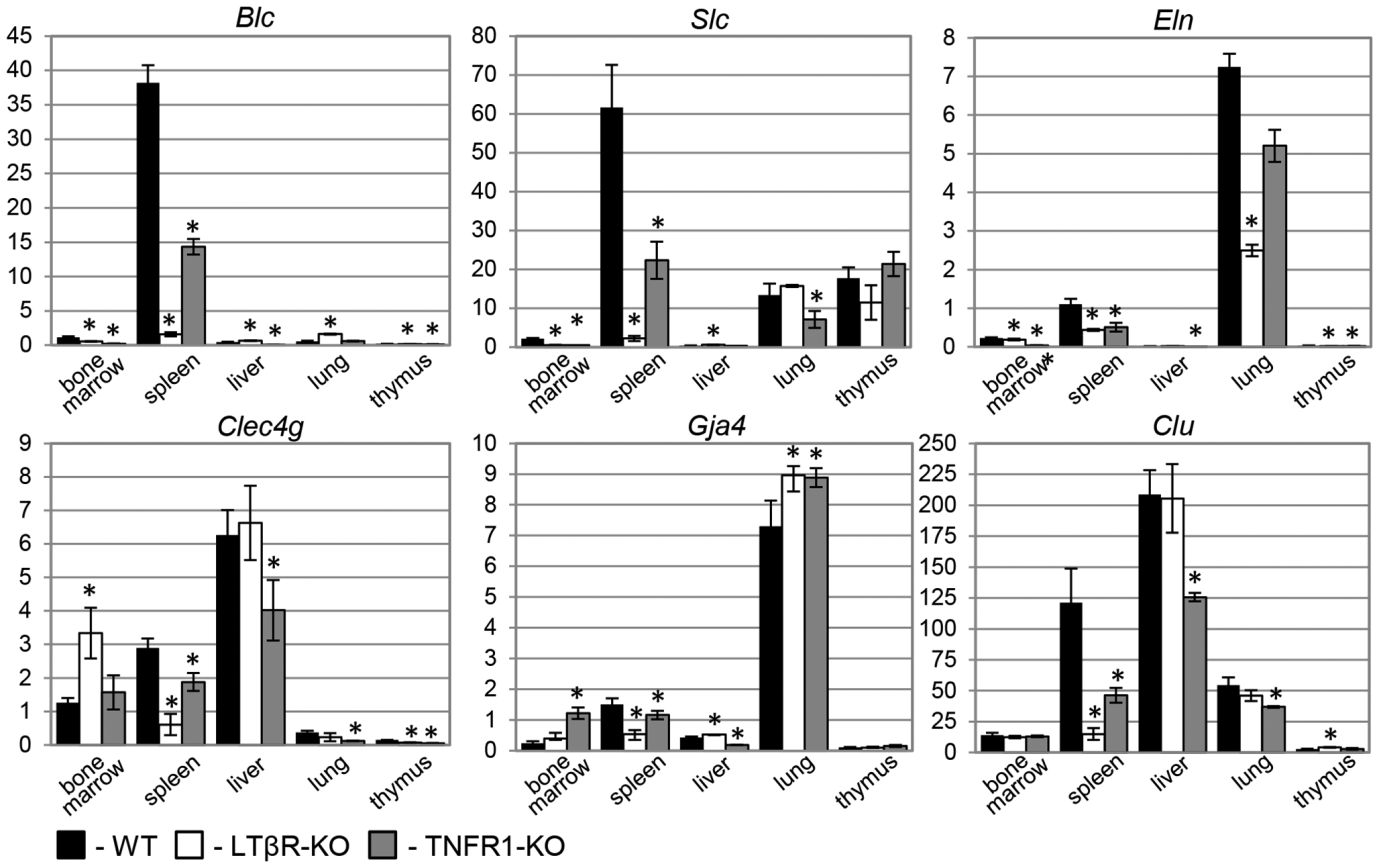
Relative mRNA levels of known and potential LTβR target genes in various mouse tissues. Real-time PCR data on mRNA levels in various tissues from wild type, TNFR1-KO, and LTβR-KO mice of selected genes down-regulated in LTβR-KO splenic stroma. Data was normalized to GAPDH, which expression level was taken as 100%. Note high expression of clusterin in wild type spleen and its dramatic reduction in spleen upon LTβR knockout. Data is represented as mean±SD. * – Difference from the wild type is significant at *p*<0.05.

We compared transcriptomes of freshly isolated LTβR-KO and WT splenic stroma (see Materials and Methods for details) and identified 505 genes (Table S1) with predominantly stromal expression, which showed >1.5-fold reduction of mRNA level upon LTβR knock-out (further designated as “potential LTβR target”, or “PLT” genes). Microarray results were confirmed by quantitative PCR and Northern blot analyses (fig. 1 and data not shown). Among PLT genes are a number of known targets of LTβR: *Madcam1* [*23*] *, Cxcl13(Blc), Ccl21a(Slc), Vegfa* [*24*], *Prnp* [*25*], which confirms the validity of our approach. Some of the known LTβR targets such as *Ccl19, Baff* and *Sdf1*, could not be assessed with the array used for technical reasons. *Il7* appeared to be downregulated in LTβR-KO spleen in agreement with its expression by gp38-positive stromal cells [26], which mature under LT control [27]. Somewhat surprisingly, *Vcam1* expression in splenic stroma was not affected by LTβR knock-out, even though this gene was previously shown to be directly activated by LTβR stimulation via canonical NFkB pathway [28], [29]. 24 genes showed >1.5-fold elevated expression in LTβR-KO spleen as compared to wild type (Table S2), indicating that these genes may be negatively regulated by LTβR signaling pathway.

Strikingly, spleen stroma cultured for 3–5 weeks in the absence of lymphocytes dramatically changed its expression profile. Cluster analysis revealed that transcriptome of cultured splenic stroma is the most distant one among all the studied groups of samples (Figure S1).

Endothelial and smooth muscle cells of white pulp express LTβR, and its activation is required to maintain proper marginal sinus vascular structure and function [30]. In accordance with that, *Gap Junction Protein Alpha 4*, *Endothelin 1* and other genes of vasculature development cluster were highly enriched among PLT genes (Enrichment Score: 6.43, *p-values* for individual annotation terms ≤3.4•10^−6^).

Interestingly, nervous system-related genes also appeared to be highly enriched in PLT gene group (Enrichment Score: 3.2, *p-values*≤0.006 for neurogenesis cluster; Enrichment Score: 1.94, *p-values*≤0.026 for regulation of neurotransmitter secretion cluster). That may be of physiological significance, since innervation of SLOs was shown to be important for their function [31]. Distribution of nerve fibers of different types in spleen of LTβR-KO mice has not been studied yet, but represents an interesting question.

### *Clusterin* gene expression is dependent on LTβR signaling

LTβR knock-out results in a significant decrease in *Clu* gene expression in splenic stroma (by the factor of 3 according to microarray data and by almost an order of magnitude according to quantitative real-time PCR) (Table S1 and fig. 1). This is in accordance with previously reported *Clu* downregulation in mouse spleen upon combined lymphotoxin-α and TNF knock-out [32] as well as with the fact that *Clu* transcripts are significantly overrepresented in FDC-enriched cell fraction of mouse spleen and are consistently down-regulated in soluble LTβR-Ig-treated mesenteric lymph nodes [5]. Among studied organs (bone marrow, spleen, liver, lung and thymus), dependence of *Clu* mRNA level on LTβR expression was seen only in spleen, where it also depended on the presence of TNFR1 but to a lesser extent. Interestingly, relationship between splenic *Clu* mRNA levels in WT, LTβR-KO and TNFR1-KO mice was very similar to that of two well-studied LTβR targets *Blc* and *Slc* (Fig. 1).

In order to demonstrate more directly that *Clu* expression can be activated by LT, we incubated MEF with Reh human B-lymphocytic leukemia cells. Reh cells were shown to constitutively express high amounts of LT heterotrimer on their surface without expressing TNFα [33], and human LT was shown to effectively interact with the murine LTβR receptor [34]. *Blc* and *Vcam1* mRNAs, previously shown to peak in response to LTβR cross-linking in MEF at 24 h and 3 h, respectively [2], [35], were used as controls for proper activation. We used Jurkat human T-cell line as a negative control, since flow cytometry showed the absence of surface LT epitopes on these cells [33].

**Figure 2:**
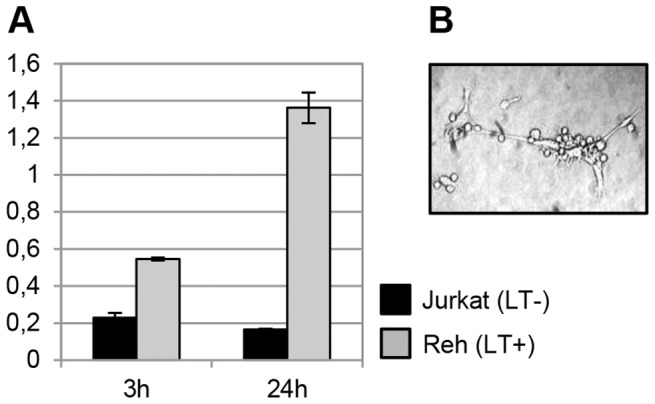
Clusterin expression in activated MEF. (A) MEF were incubated with either Reh cells bearing surface LT (“LT+”) or Jurkat cells not expressing LT on their surface (“LT−”) for indicated time periods, and clusterin mRNA was measured by real-time RT-PCR. Data was normalized to mouse β-actin. (B) Physical interaction of MEF with lymphoid cells in culture. Data is represented as mean±SD.

Cocultivation of MEF with Reh but not Jurkat cells for 24 h significantly induced *Clu* mRNA (fig. 2). Temporal dynamics of *Clu* upregulation resembled that published for *Blc*, but not *Vcam1* [2], [28], indicating involvement of alternative rather than classical NFκB pathway in *Clu* activation via LTβR.

### Splenic CLU isoform and its distribution among stromal cell subpopulations

There are several CLU protein isoforms encoded by two CLU gene transcripts. The main and longer gene transcript encodes glycosylated presecretory form psCLU with apparent molecular weight of about 60 kDa. Cleavage into α- and β-chains and further extensive glycosylation produces a mature, secreted heterodimeric 70–80 kDa protein referred to as sCLU. Under reducing conditions both α- and β-subunits of sCLU run at about 40 kDa at SDS-PAGE. The second transcript lacks the endoplasmic reticulum-targeting sequence at exon 2 and its product is detected as 49 kDa non-glycosylated pnCLU precursor in the cytosol and a 55-kDa glycosylated nCLU protein in the nucleus [36].

**Figure 3:**
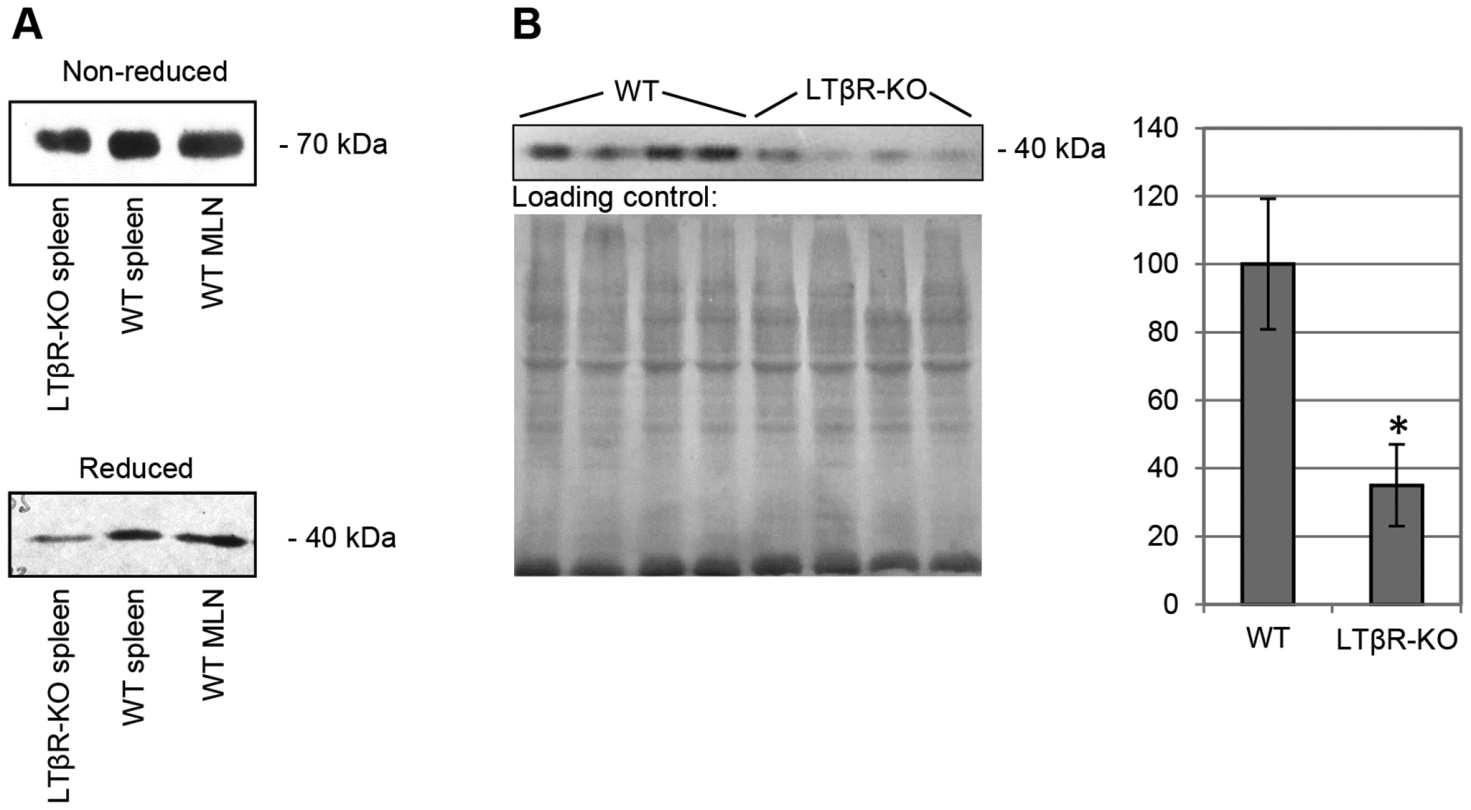
Western blot analysis of CLU isoform. (A) Electrophoresis of stromal proteins from spleen and mesenteric lymph nodes (MLN) was performed in reducing and non-reducing conditions. Immunopositive bands mobility corresponds to secreted CLU isoform (sCLU). (B) Quantitative comparison of splenic sCLU expression in wild type (WT) and LTβR-deficient (LTβR-KO) mice. Data is normalized to the average WT expression and represented as mean±SD. * – Difference from the wild type is significant at *p*<0.05.

Secretory and nuclear forms of clusterin are considered to have somewhat opposing functions, with sCLU being a cell-protective, anti-apoptotic protein, and nCLU acting as a pro-death signal, inhibiting cell growth and survival [37]. As it is important for understanding the clusterin functions in SLO, we assessed CLU protein isoform in the splenic stroma using Western blot. Clusterin immunopositive band ran around 70 kDa in non-reducing conditions, and around 40 kDa in reducing conditions, which corresponds to sCLU and its two co-migrating subunits, respectively (fig. 3a). The pattern was similar for WT and KO mice, however the intensity of CLU bands in KO mice was significantly reduced (fig. 3b).

In order to assess cellular distribution of sCLU in splenic stroma, we used immunohistochemical staining of frozen spleen sections with commercial polyclonal anti-CLU antibodies (R&D AF2747) raised against recombinant mouse CLU Glu22-Glu448. Polyclonality and usage of almost full-length protein as immunogen ensured that this antibody would recognize different CLU isoforms in different applications. AF2747 specificity was confirmed by specific staining of HEK293 cells transiently transfected with full-length CLU (data not shown).

**Figure 4:**
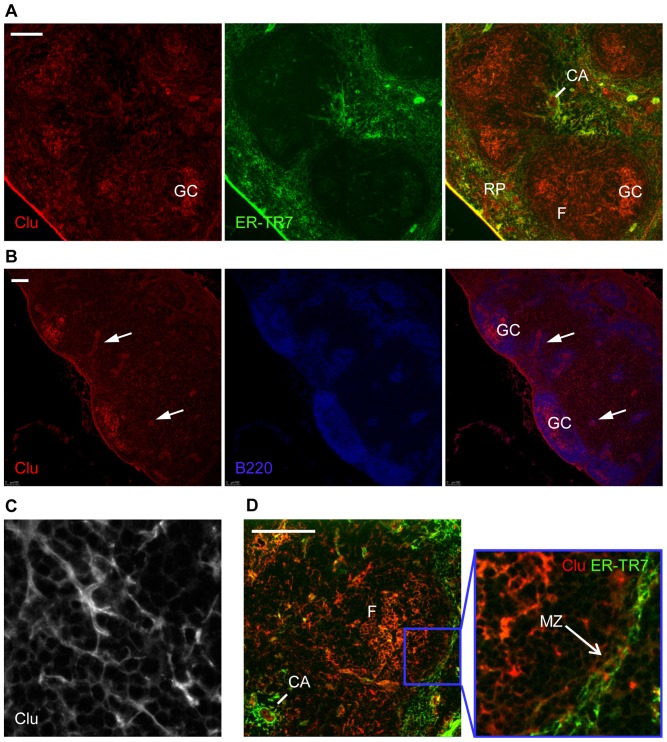
Cellular and tissue distribution of clusterin. Immunohistochemical staining of wild type spleen (A) and MLN (B) after immunization with SRBC. (C) Shows an image of clusterin-positive spleen stromal cell under high power (original magnification 630×), showing that clusterin is located near cell membrane. (D) Double staining for MRC marker ER-TR7 (green) and clusterin (red). Ubiquitous presence of clusterin-positive cells can be seen in all stromal compartments except for splenic marginal zone (D), with the brightest staining seen in germinal centers. Scale bar: 100 µm. GC – germinal center, CA – central arteriole, RP – red pulp, F – follicle, arrows – high endothelial venules, MZ – marginal zone. Data is representative of at least 2 experiments.

**Figure 5:**
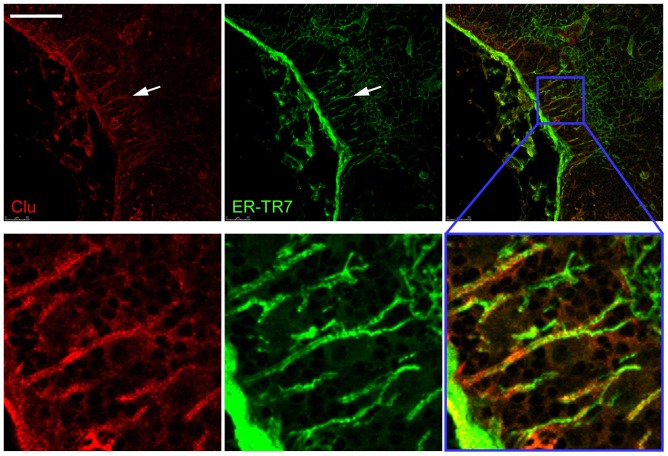
Interfollicular channel region of mouse mesenteric lymph node. MLN was stained with anti-clusterin and ER-TR7 antibodies after immunization with SRBC. Note clear immunopositivity of conduits (arrows) for clusterin. Lower row represents the close up of the indicated square region. Data is representative of at least 2 experiments. Scale bar: 100 µm.

Multi-color immunostaining with B-220 (recognizing B-cells) and ER-TR7 (recognizing fibroblastic reticular cells, marginal reticular cells and red pulp fibroblasts) showed that clusterin was expressed by all subsets of stromal cells in spleen and mesenteric lymph nodes (MLN) except for marginal reticular cells (MRC) (fig. 4, 5). This expression pattern is broader than previously reported [5], [19], [38], though the brightest staining was still observed in B-cell areas, especially in GCs after immunization, and is attributed to FDC for which clusterin is used as one of differential markers [38], [39]. An important difference with the previous observations consists in the clear absence of marginal zone staining in spleen (fig. 4d). Diffuse staining was observed in spleen red pulp, MLN medulla and lumen of high endothelial venules, which can be explained by the high amount of sCLU in blood [13]. GC staining also had a diffuse appearance, not resembling stromal cell contours, which may be indicative of active secretion of sCLU in this area. Previously, sCLU secretion by FDC was shown by Verbrugghe et al. who detected clusterin immunoreactivity in the endoplasmic reticulum, Golgi apparatus, and on the plasma membrane of FDC in human Payer's patches by electron microscopy [18].

In contrast to the wild type pattern, only faint staining of few stromal cells could be seen in disorganized white pulp of the spleens of LTβR-KO mice (data not shown). Diffuse staining of red pulp was not affected. This may reflect not only the absence of FDC, which contribute to the bright staining of B-cell follicles in WT mice spleen, but also downregulation of CLU in other stromal cell types in the absence of LTβR signal.

### sCLU dynamics during immune response

**Figure 6:**
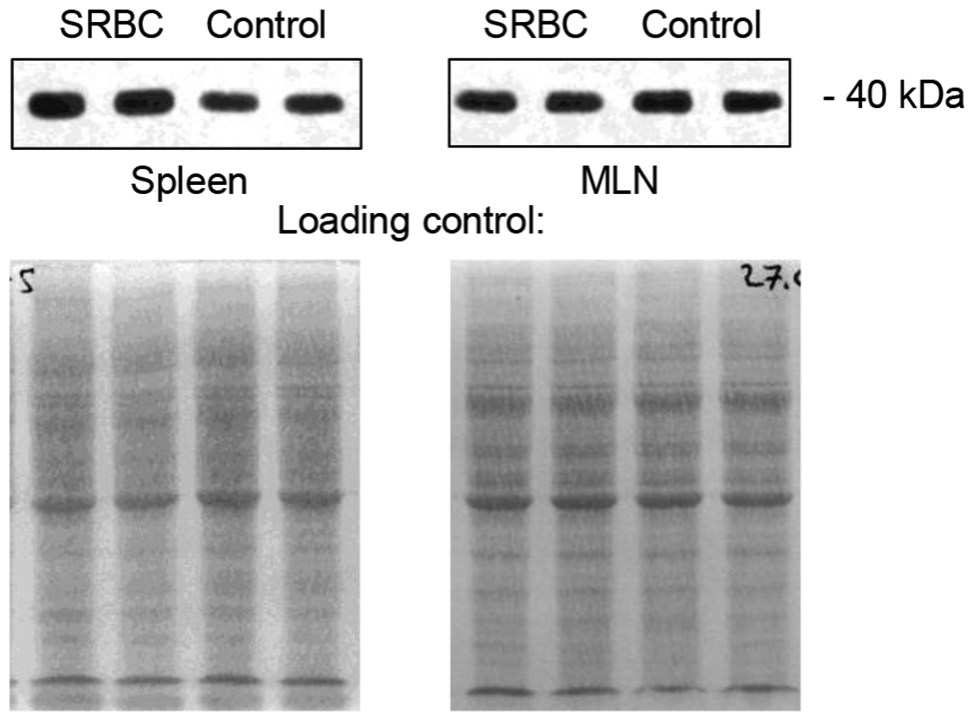
Changes in sCLU protein level in spleen and MLN of WT mice at day 8 after immunization with SRBC. Western blot of total tissue homogenates shows an increase in sCLU amount in spleen but not MLN. Data is representative of 2 independent experiments.

**Figure 7:**
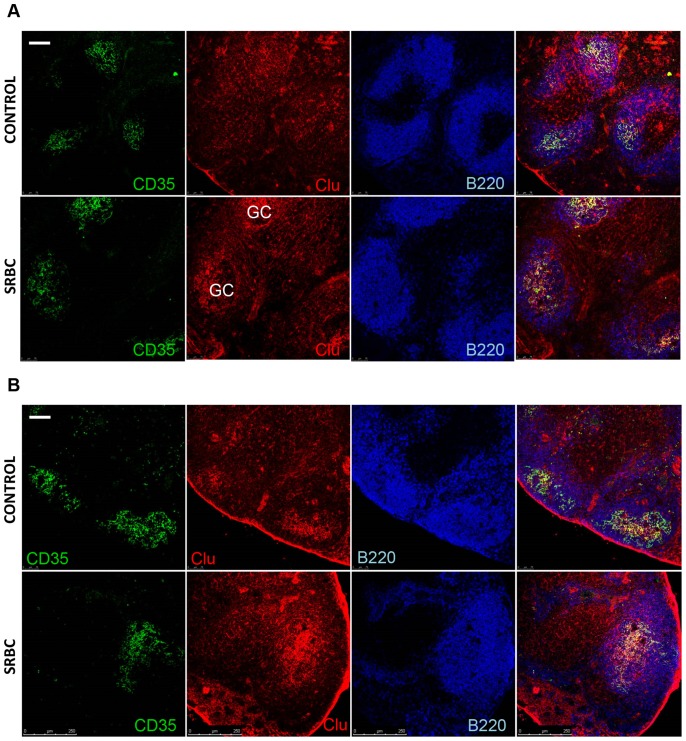
Changes in sCLU tissue distribution in spleen and MLN of WT mice at day 8 after immunization with SRBC. (A) Immunohistochemical staining of WT spleen cryosections showing accumulation of clusterin in the light zones of germinal centers (GC) upon immunization. (B) No changes in clusterin tissue distribution and quantity can be seen in MLN of WT mice after SRBC immunization. Data is representative of at least 2 independent experiments. Scale bar: 100 µm.

CLU was previously shown to be induced during tissue remodeling in mammary gland [40]. Its mRNA and protein levels substantially rise during pregnancy, when mammary tissue undergoes structural changes, and at the early stages of post-weaning involution accompanied by high rates of apoptotic death [11]. This data and also the fact that CLU may serve as a survival factor for GC B-cells [5] prompted us to study CLU protein dynamics in spleen and MLN during immune response by immunohistochemistry and Western blot. The development of GC reaction was confirmed by peanut agglutinin (PNA) staining. Only in spleen, but not in MLN, CLU protein level was elevated at the peak of GC formation (fig. 6). This overall increase was accompanied by the accumulation of CLU immunoreactivity in the light zones of GCs (fig. 7a). That was not the case in MLN, where bright focuses of CLU could be seen in centers of B-cell follicles independently of immunization and GC presence (fig. 7b). No parallel increase in *Clu* mRNA level could be detected in spleen by quantitative PCR (data not shown) indicating that either *Clu* mRNA peaks before the 8th day post immunization, or CLU protein synthesis is controlled at the post-transcriptional level.

## Materials and Methods

### Ethics Statement

This study was carried out in strict accordance with the recommendations in the Guide for the Care and Use of Laboratory Animals (NRC 2011), the European Convention for the Protection of Vertebrate Animals Used for Experimental and Other Scientific Purposes, Council of Europe (ETS 123), and “The Guidelines for Manipulations with Experimental Animals” (the decree of the Presidium of the Russian Academy of Sciences of April 02, 1980, no. 12000-496). All manipulations with animals were approved by Scientific Council of the Engelhard Institute of Molecular Biology. The Three Rs Principle was implemented as follows: a) replacement: at present, it is impossible to perform the proposed biochemical and physiological experiments using cell line models. The initial key observation was done by *ex vivo* utilization of organs of untreated mice for microarray experiments. However the cultured splenic stroma has dramatically changed the expression profile, thereby couldn't be utilized for further LTβR regulated genes/proteins studies *in vitro*. The use of primary MEFs co-culture with lymphoid cell lines Reh and Jurkat can only partially replace *in vivo* experiments, and won't be suitable for histological studies. To perform *ex vivo* organ examination and primary cultures all efforts were made to ameliorate animal suffering. Animal sacrifice was performed by CO_2_ asphyxiation, checked for responsiveness and followed by cervical dislocation for adult mice; b) reduction: animal studies are preceded by multiple experiments *ex vivo*, *in vitro* or in primary cell cultures *in vitro*. Minimum number of animals was used in each experiment to achieve statistically significant data (n = 3 or 4). The isolation of spleens and lymph nodes was combined from each animal and parts of the same organs were used for histology, Western blot and real-time PCR to keep the number of required animals to an absolute minimum; c) refinement: Animal studies were only used at advanced stages of investigations when few, specific and highly relevant questions – such as clusterin bio-distribution upon immunization – were addressed by a limited number of experiments.

### Animals and Housing Conditions

We obtained 8–10-week-old inbred mice (weight 20–25 g) C57BL/6, LTβR-KO [22] (mice deficient for lymphotoxin β receptor), and TNFR1-KO [41] (mice deficient for tumor necrosis factor receptor type 1) mice from the stock of the specific pathogen free Laboratory Animal Breeding Facility “Pushchino” (http://spf-animals.ru/). The knockout mice were backcrossed to C57BL/6 more than 10 times. All experimental animals were housed according to “Recommendations for the health monitoring of mouse, rat, hamster, gunea pig and rabbit breeding colonies” FELASA (Federation of European Laboratory Animal Science Associations) recommendations, June, 2001. All mice were housed (for 4–6 weeks) in single-sex groups, of 3 or 5 in a cage. Male and female groups were maintained in standard plastic cages (35×21×9 cm) with sawdust (wooden flakes) as nesting material. Food pellets (BioPro, Novosibirsk) and water were provided *ad libitum*. Mice were maintained at 22–24°C under 12∶12 light-dark cycle with lights off at 9.00 p.m.. Every 5 days, animals were placed into a clean cage with fresh sawdust. In each cage, mice were individually marked by ear cuts. When C57/BL6 mice were used as controls they were age and sex matched to the KO mice and co-housed for a minimum of two weeks, in the most cases littermates were used as controls.

### Mice immunizations

For immunization, 8 week-old mice were injected intraperitoneally in their home cages with 2•10^8^ SRBC in 300 µl of sterile PBS. Control littermate animals were left intact. Mice were sacrificed 8 days after injection on the pick of germinal center reaction, and mesenteric lymph nodes and spleens were immediately isolated and put on ice in RPMI medium until further manipulations. 3 mice of each genotype: C57Bl6 and LTβR-KO (overall 2 experimental groups), were immunized with SRBCs; organs from 3 untreated mice of each genotype were used as a respective controls for histological studies or Western blot. Immunization and analysis were repeated at least 2 times. Thereby, the total mice number was N = 24, where C57BL/6 n = 12 and LTβR-KO n = 12. We utilized 3 mice of each genotype or group per experiment as minimum sample size sufficient for statistical analysis, however the statistical analysis of histological score and/or counts of certain stained objects on several fields of microscopic view are not presented, since the observed clusterin distribution was clearly reproduced in several independent experiments.

### Splenic stromal cell culture

Spleens of 8–10 week-old C57BL/6 mice were aseptically isolated, grinded with sterile scissors in a Petry dish and cultured in DMEM (Paneco, Moscow, Russia) supplemented with 10% FBS (Biological Industries, Kibbutz, Israel), 2 mM L-glutamine (HyClone), MEM Non-Essential Amino Acids Solution (HyClone), 0.1 mM Sodium Piruvate (HyClone), 10 mM HEPES (HyClone), 100 U/ml Penicillin 100 ug/ml Streptomycin (Gibco). After 5–7 days of cultivation non-adherent cells were removed by 3 washes with fresh medium. Remaining cells were trypsinized and replated weekly beginning from day 14 after isolation. Cells were used for experiments at week 3–4 of cultivation.

### Preparation of samples and microarray hybridization

For microarray hybridization, four types of samples were prepared: uncultured splenic stroma of C57BL/6 mice, uncultured splenic stroma of LTβR-KO mice, splenocytes of C57BL/6 mice and cultured splenic stroma of C57BL/6 mice. To obtain splenocyte- and stroma-enriched fractions, freshly isolated spleens were rubbed over 70 µm mesh and washed with PBS on ice. The wash, containing splenocytes, was collected, centrifuged, and the pellet was homogenized in TRIzol (Life Technologies). Remaining stroma was collected from the mesh, cut with scissors and homogenized in TRIzol. Total RNA isolation, amplification and hybridization on Illumina chip were performed by ZAO “Genoanalytica”. Briefly, total RNA was extracted from the samples according to TRIzol manufacturer's instruction. RNA was quantified using Nanodrop and its quality was assessed by Agilent Total RNA 6000 chip. 400 ng of total RNA was amplified by Illumina TotalPrep RNA Amplification Kit (Ambion). Amplified RNA was hybridized with MouseRef-8 v1.1 Expression BeadChips (Illumina) according to Illumina protocol.

### Microarray data acquisition and analysis

Microarray data acquisition and analysis were done with GenomeStudio Gene Expression Module v1.0 (Illumina) (accession number in the NCBI GEO database is GSE52294). After normalization between samples and replicates using «cubic spline» method [42] and background substitution, 1542 genes were selected on the basis of differential p value <0.05 when comparing wild type with knock-out stroma (p values were calculated using “Illumina Custom” algorithm for multiple comparisons with Benjamini–Hochberg procedure controlling the false discovery rate). To avoid artifacts resulting from splenocyte contamination of stromal preparations, only genes with expression levels in C57BL/6 stroma greater than in C57BL/6 splenocytes were considered for further analysis. Functional annotation clustering of differentially expressed genes was performed using DAVID analytic tools [43]. Customized parameters were: Annotation categories - GOTERM_BP_4, Classification stringency - medium, Enrichment Thresholds - EASE = 0.05.

### Quantitative real-time PCR

Entire organs or isolated stroma were first cut with scissors on ice. Resulting material, bone marrow aspirates or cultured cells were homogenized in TRIzol reagent by pipetting. Total RNA was isolated according to TRIzol manufacturer's instructions. The RNA quality was assessed by spectrophotometry and agarose gel electrophoresis. 4 µg of the total RNA was taken for cDNA synthesis using RevertAid First Strand cDNA Synthesis Kit (Thermo Scientific) according to manufacturer's protocol. Quantitative real-time PCR was performed using EVA Green Real-Time PCR kit (Syntol, Moscow, Russia) and 7500 Real-Time PCR System (Applied Biosystems). Primer sequences were: *Clu*, 5′ - CTGTCCACTCAAGGGAGTAGG and 5′ - GTGTCCTCCAGAGCATCCTC; *Blc*, 5′ - CATAGATCGGATTCAAGTTACG and 5′ - TCTTGGTCCAGATCACAACTTC; *Clec4g*, 5′ - TACTGTCCAGTGCCTCCAGCAAG and 5′ - TGTCACGGAGCAGCAATTCCTG; *Gja4*, 5′ - GCAAGCAGGCGAGAGAGG and 5′- AGATGAAGAGCACCGTTAACCAG; *Hmgcs2*, 5′ - GGTGTCCCGTCTAATGGAGA and 5′ - ACACCCAGGATTCACAGAGG; *Slc*, 5′ - ATGGCTCAGATGATGACTCTG and 5′ - TAGCCTCGGACAATACTGTAGG. For positive control and normalization, *β-actin* primers discriminating mouse from human were used: 5′ - CCGCGAGCACAGCTTCTTTG and 5′ - CCATCACACCCTGGTGCCTA. Forward and reverse primers were complimentary to different exons so that they did not give any products using genomic DNA as a template.

### Western blot

Entire spleens and MLNs were cut with scissors on ice, homogenized in ice-cold RIPA buffer (150 mM NaCl, 1% NP-40, 0.1% SDS, 50 mM Tris buffer, pH 8.0) by gentle sonication, and clarified by centrifugation at 12000 g for 10 min at +4°C. Total protein concentration was measured in supernatants by Bradford protein assay. 5× Laemmli buffer with or without β-mercaptoethanol was then added 1∶4 (v/v) and samples were boiled for 5 min. After separation by SDS-PAGE electrophoresis (20 µg of total protein per well) proteins were transferred to Hybond-C Extra nitrocellulose membrane (Amersham Biosciences) using Mini Trans-Blot system (Bio-Rad). Uniform loading and transfer was confirmed by Ponceau Red staining. Membranes were blocked with 3% BSA in TBST for 30 min, stained with anti-clusterin antibody (1∶3000; AF2747 R&D Systems) overnight at +4°C, washed with TBST, and incubated with secondary HRP-conjugated anti-goat antibody (1∶50000; Pierce) in 5% NFDM TBST solution for 1 h at room temperature. After membranes were thoroughly washed in TBST, specific bands were visualized by SuperSignal West Dura Extended Duration Substrate (Thermo Scientific) and x-ray film. Densitometry was performed using ImageJ software (NIH, USA).

### Immunofluorescence

Fresh spleens and MLNs were embedded in O.C.T. Compound (Sakura) and frozen at −60°C. 10 µm cryosections were fixed with acetone (70% for 2 min followed by 100% for 8 min). Before staining, slides were incubated in blocking solution (5% rabbit serum, 1.4 µg/ml anti-mFcγR, 0.1% Triton X-100 in PBS) for one hour at room temperature. Primary antibodies (Abs) were diluted in PBS containing 1% rabbit serum, 1% BSA, 0.3% Triton X-100, 2.8 µg/ml anti-mFcγR; secondary Abs were diluted in PBS containing 5% rabbit serum, 0.1% Triton X-100, 2.8 µg/ml anti-mFcγR. Staining was performed overnight at +4°C for anti-clusterin Abs, and for one hour at room temperature for the other Abs. The following Abs were used: Goat anti-clusterin (1∶40; R&D Systems), Rat ER-TR7 (1∶300; BMA Biomedicals), Rat anti-CD35-Bio (1∶300; BD Pharmingen), Rat anti-B220-Cy5 (1∶300; Abnova); PNA-FITC (1∶300; VectorLabs), Streptavidin-FITC (1∶300; BD Pharmingen), Donkey anti-Goat IgG(H+L)-Alexa 594 (1∶1000; Invitrogen). Images were obtained with Leica TCS SP5 Confocal Microscope and Leica Application Suite software.

### MEF activation

MEFs were prepared from 13.5-day-old fetuses of C57BL/6 wild type mice. All procedures were approved by the Scientific Council of the Engelhard Institute of molecular biology, according to “The Guidelines for Manipulations with Experimental Animals” (the decree of the Presidium of the Russian Academy of Sciences of April 02, 1980, no. 12000-496). To obtain embryos all efforts were made to ameliorate animal suffering. Adult mice sacrifice was performed by CO2 asphyxiation, checked for responsiveness and followed by cervical dislocation. Embryos were separated from the placenta and surrounding membranes. The brain and dark-red organs were dissected and discarded. Remaining body parts were rinsed with DMEM, dissected into small pieces and treated with 1 mL of trypsin/EDTA at 37°C for 20 min to separate cells. After addition of 6 mL of fresh medium, cells were further separated by pipetting and put to CO_2_ incubator overnight. The next day non-adherent cells were removed during medium replacement. Adherent cells were let to reach confluence for 1–3 days. After consequent trypsinization and centrifugation only single-cell fraction was used for further replating. MEF were cultured in DMEM (Paneco, Moscow, Russia) supplemented with 10% FBS (Biological Industries, Kibbutz, Israel), 2 mM L-glutamine (HyClone), MEM Non-Essential Amino Acids Solution (HyClone), 0.1 mM Sodium Piruvate (HyClone), 10 mM HEPES (HyClone), 100 U/ml Penicillin 100 ug/ml Streptomycin (Gibco). At the second passage MEF were plated after trypsinization onto 6 cm dishes, 5×10^5^ cells per dish. Next morning, the medium was removed and 8 ml of fresh RPMI containing 5×10^6^ Reh or Jurkat cells was added for LTβR activation. After 3 and 24 hours, MEF were washed twice with PBS and homogenized in 1 ml of TRIzol reagent for further RNA isolation. *Vcam1* and *Blc* expression levels were assessed by semi-quantitative PCR (primer sequences were: *Blc*, 5′- CAGAATGAGGCTCAGCACAGC and 5′- TGCAACGGAGCTTGAGCATTCC; *Vcam1*, 5′- AATGCCTGTGAAGATGGTCG and 5′- GAACAGGTCATTGTCACAGC) as a control for proper activation.

### Statistical analysis

Statistical significance of differences between groups was tested with the Mann–Whitney U test using Statistica 7.0 software (StatSoft, Inc.). All data are expressed as mean ±SD.

## Supporting Information

Figure S1 Cluster analysis of the microarray data.
(DOCX)

Table S1 The list of genes which mRNA levels were more than 1.5-fold higher in wild type spleen stroma comparing to LTβR-KO spleen stroma.
(DOCX)

Table S2 The list of genes which mRNA levels were more than 1.5-fold lower in wild type spleen stroma comparing to LTβR-KO spleen stroma.
(DOCX)
