# Supplementary material for: Clusterin Is a Potential Lymphotoxin Beta Receptor Target That Is Upregulated and Accumulates in Germinal Centers of Mouse Spleen during Immune Response
Source: PLoS One. 2014 May 27;9(5):e98349. doi: 10.1371/journal.pone.0098349 (PMC4035297; doi:10.1371/journal.pone.0098349)
Supplement: Figure S1 — Cluster analysis of the microarray data. (DOCX) [file pone.0098349.s001.docx]

**Figure S1**

Cluster analysis of the microarray data. Two biological replicates of each sample type were analyzed with Illumina beadchip.
