# Supplementary material for: Clusterin Is a Potential Lymphotoxin Beta Receptor Target That Is Upregulated and Accumulates in Germinal Centers of Mouse Spleen during Immune Response
Source: PLoS One. 2014 May 27;9(5):e98349. doi: 10.1371/journal.pone.0098349 (PMC4035297; doi:10.1371/journal.pone.0098349)
Supplement: Table S1 — The list of genes which mRNA levels were more than 1.5-fold higher in wild type spleen stroma comparing to LTβR-KO spleen stroma. (DOCX) [file pone.0098349.s002.docx]

**Table S1**

The list of genes which mRNA levels were more than 1.5-fold higher in wild type spleen stroma comparing to LTβR-KO spleen stroma. Known LTβR targets are shown in **bold.**

| Gene name | WT:LTβR-KO ratio |
| --- | --- |
| *1500015O10Rik* | 2.5 |
| *1700019E19Rik* | 1.5 |
| *1700029G01Rik* | 2.0 |
| *2310022B05Rik* | 1.9 |
| *3321401G04Rik* | 1.9 |
| *4833430A08Rik* | 2.0 |
| *4931432E15Rik* | 4.9 |
| *5330431K02Rik* | 2.1 |
| *5430407P10Rik* | 2.6 |
| *9030224M15Rik* | 3.8 |
| *9530068E07Rik* | 1.8 |
| *Aatk* | 1.7 |
| *Abhd14b* | 1.5 |
| *Accn2* | 3.2 |
| *Acta2* | 1.6 |
| *Acta2* | 1.7 |
| *Actb* | 1.6 |
| *Actg2* | 2.0 |
| *Acvr2b* | 1.6 |
| *Adam11* | 2.1 |
| *Adam15* | 1.8 |
| *Adam33* | 4.0 |
| *Aebp1* | 1.8 |
| *Agrn* | 1.9 |
| *Aif1l* | 4.1 |
| *Alas1* | 1.6 |
| *Aldh3a1* | 3.6 |
| *Alg2* | 1.6 |
| *Alox12* | 1.6 |
| *Alpl* | 2.3 |
| *Amotl1* | 1.7 |
| *Anxa3* | 1.7 |
| *Aoc3* | 2.5 |
| *Apbb1* | 2.5 |
| *App* | 1.6 |
| *Arap3* | 1.6 |
| *Arhgap32* | 1.8 |
| *Arhgap44* | 2.2 |
| *Arhgef10l* | 2.0 |
| *Arhgef25* | 1.9 |
| *Asah2* | 1.8 |
| *Asb2* | 2.3 |
| *Atp6v1g2* | 1.6 |
| *AW555464* | 1.8 |
| *Azi1* | 2.1 |
| *B3gnt1* | 1.8 |
| *B930041F14Rik* | 1.6 |
| *B930095G15Rik* | 1.9 |
| *Bace2* | 1.5 |
| *Bahcc1* | 2.7 |
| *BC034090* | 1.7 |
| *Bik* | 2.1 |
| *Bok* | 1.6 |
| *C130074G19Rik* | 1.8 |
| *C530043K16Rik* | 2.0 |
| *Camk2a* | 2.3 |
| *Car11* | 1.7 |
| *Card10* | 2.0 |
| *Ccdc3* | 2.2 |
| ***Ccl21a*** | 10.4 |
| *Ccnd1* | 2.1 |
| *Cd209b* | 6.0 |
| *Cd300lg* | 2.5 |
| *Cd33* | 5.6 |
| *Cdc42ep2* | 1.6 |
| *Cdh13* | 2.2 |
| *Cdh3* | 3.9 |
| *Cdhr1* | 4.6 |
| *Cdk20* | 2.4 |
| *Cds1* | 1.7 |
| *Ch25h* | 2.4 |
| *Chst1* | 3.6 |
| *Ckb* | 2.1 |
| *Clec11a* | 1.9 |
| *Clec4g* | 6.3 |
| *Clec9a* | 2.2 |
| *Clmp* | 1.7 |
| *Clu* | 3.2 |
| *Cmtm3* | 1.5 |
| *Cobl* | 2.1 |
| *Col16a1* | 1.7 |
| *Col18a1* | 1.6 |
| *Col20a1* | 1.5 |
| *Col5a1* | 1.6 |
| *Col6a1* | 2.0 |
| *Col6a2* | 1.6 |
| *Copz2* | 1.7 |
| *Corin* | 3.2 |
| *Cpe* | 2.2 |
| *Crip2* | 2.2 |
| *Cryab* | 1.8 |
| *Cryba4* | 1.7 |
| *Csf1* | 1.6 |
| *Csnk1e* | 1.9 |
| *Csrp2* | 1.7 |
| *Cx3cl1* | 2.8 |
| *Cx3cr1* | 3.0 |
| ***Cxcl13*** | 3.7 |
| *Cxcr7* | 2.2 |
| *Cyp2d22* | 1.6 |
| *D0H4S114* | 2.4 |
| *D4Bwg0951e* | 3.0 |
| *Dab2* | 1.8 |
| *Dab2ip* | 2.0 |
| *Dbn1* | 2.2 |
| *Dbndd2* | 1.7 |
| *Dcakd* | 1.8 |
| *Ddah2* | 1.8 |
| *Dennd3* | 1.8 |
| *Des* | 2.3 |
| *Dgkg* | 2.8 |
| *Dll1* | 2.2 |
| *Dnajb5* | 1.8 |
| *Dnajc6* | 2.1 |
| *Dnase1l3* | 2.5 |
| *Dok4* | 1.9 |
| *Dram1* | 2.5 |
| *Dusp23* | 2.1 |
| *Dzip1l* | 1.8 |
| *Edem2* | 1.6 |
| *Edn1* | 3.4 |
| *Ednrb* | 1.7 |
| *Efna1* | 1.7 |
| *Efna5* | 2.1 |
| *Efnb1* | 2.9 |
| *Ehd1* | 2.1 |
| *Ehd4* | 1.7 |
| *Elmo2* | 4.0 |
| *Eln* | 4.1 |
| *Emcn* | 2.7 |
| *Eml1* | 2.0 |
| *Emp2* | 1.7 |
| *Enpp2* | 6.6 |
| *Entpd2* | 1.7 |
| *Epha8* | 2.1 |
| *Ephx3* | 6.5 |
| *Erbb2* | 1.9 |
| *Erdr1* | 157.6 |
| *Eri3* | 1.7 |
| *Esam* | 2.3 |
| *Esm1* | 2.5 |
| *Etl4* | 1.8 |
| *Evc2* | 1.7 |
| *Exoc3l4* | 2.5 |
| *Exoc6b* | 1.5 |
| *Extl1* | 4.9 |
| *F2rl3* | 1.5 |
| *Fam110b* | 2.0 |
| *Fam129b* | 2.0 |
| *Fam13c* | 1.7 |
| *Fam164a* | 1.5 |
| *Fam54b* | 1.6 |
| *Farp1* | 1.5 |
| *Fbln2* | 1.9 |
| *Fbxl16* | 3.7 |
| *Fbxw17* | 1.6 |
| *Fermt2* | 1.7 |
| *Fes* | 1.6 |
| *Fez1* | 3.2 |
| *Fez1* | 3.2 |
| *Fgf1* | 3.0 |
| *Fhl1* | 2.2 |
| *Fkbp10* | 1.8 |
| *Flnc* | 2.3 |
| *Flot1* | 1.6 |
| *Flrt3* | 7.2 |
| *Flt1* | 1.6 |
| *Fosl2* | 2.0 |
| *Foxs1* | 3.8 |
| *Fto* | 1.6 |
| *Fxyd6* | 1.5 |
| *Gaa* | 1.7 |
| *Galntl1* | 4.0 |
| *Galntl4* | 2.2 |
| *Garnl3* | 1.6 |
| *Gatsl3* | 1.7 |
| *Gbx2* | 3.5 |
| *Gdpd5* | 1.8 |
| *Ggt5* | 1.5 |
| *Gipc2* | 2.5 |
| *Gja4* | 3.0 |
| *Gng8* | 1.8 |
| *Golga2* | 1.6 |
| *Gpc3* | 2.1 |
| *Gpihbp1* | 1.7 |
| *Gpr125* | 1.7 |
| *Gpr176* | 1.7 |
| *Gpr20* | 5.9 |
| *Gprc5b* | 2.4 |
| *Gpsm1* | 1.5 |
| *Grn* | 1.6 |
| *Gspt2* | 1.7 |
| *Gstm1* | 1.8 |
| *Gstm2* | 1.7 |
| *Haghl* | 1.7 |
| *Hap1* | 2.9 |
| *Hey1* | 2.8 |
| *Hic1* | 1.8 |
| *Hlx* | 2.3 |
| *Hmgcs1* | 1.5 |
| *Hmgcs2* | 2.9 |
| *Hmgn3* | 1.7 |
| *Hoxb2* | 1.6 |
| *Hoxb5* | 2.1 |
| *Hs3st3a1* | 2.9 |
| *Hspa12b* | 2.6 |
| *Hspb1* | 3.0 |
| *Hspb8* | 2.0 |
| *Hspg2* | 1.9 |
| *Hspg2* | 2.1 |
| *Htra1* | 2.4 |
| *Ica1* | 1.7 |
| *Id1* | 3.2 |
| *Ier3* | 1.5 |
| *Ift81* | 2.0 |
| *Igdcc4* | 3.0 |
| *Igf2* | 1.9 |
| *Igfbp3* | 2.1 |
| *Igfbp5* | 2.1 |
| *Igfbp6* | 1.9 |
| *Il17rc* | 2.4 |
| *Il7* | 1.6 |
| *Inhbb* | 3.6 |
| *Inppl1* | 1.6 |
| *Islr* | 1.6 |
| *Itga11* | 1.7 |
| *Itga3* | 2.4 |
| *Itgb4* | 3.3 |
| *Jup* | 1.8 |
| *Kank1* | 2.0 |
| *Kcna6* | 1.9 |
| *Kcna6* | 2.0 |
| *Kcne4* | 2.0 |
| *Kcnh2* | 4.4 |
| *Kcnj8* | 1.5 |
| *Kcnk3* | 4.0 |
| *Kcnq1* | 2.8 |
| *Kctd10* | 1.8 |
| *Kctd15* | 3.3 |
| *Kdm6b* | 2.3 |
| *Kirrel* | 1.8 |
| *Klhl26* | 1.7 |
| *Lama5* | 4.6 |
| *Lamb2* | 2.1 |
| *Larp6* | 2.7 |
| *Ldb2* | 1.8 |
| *Leprel1* | 1.6 |
| *Lgi3* | 2.8 |
| *Lima1* | 1.7 |
| *Lims2* | 2.5 |
| *Lims2* | 2.7 |
| *Litaf* | 1.6 |
| *Lix1l* | 1.9 |
| *Lmcd1* | 1.8 |
| *Lmod1* | 3.3 |
| *LOC100041504* | 9.4 |
| *LOC100041504* | 10.1 |
| *LOC100047268* | 3.2 |
| *LOC100047385* | 3.1 |
| *Loxl1* | 1.7 |
| *Lpar1* | 1.7 |
| *Lpar4* | 2.2 |
| *Lpl* | 1.9 |
| *Ltbp1* | 2.6 |
| *Ltbp2* | 1.5 |
| *Ltbp3* | 1.7 |
| *Ltbp4* | 1.6 |
| *Ltc4s* | 4.2 |
| *Lyz1* | 2.6 |
| *Lzts2* | 1.8 |
| ***Madcam1*** | 43.6 |
| *Mag* | 2.1 |
| *Mapt* | 1.8 |
| *Mast4* | 1.8 |
| *Mcam* | 1.8 |
| *Mcc* | 1.6 |
| *Mcf2l* | 2.0 |
| *Mertk* | 1.5 |
| *Mfap5* | 1.5 |
| *Mfge8* | 6.7 |
| *Mgat4b* | 1.8 |
| *Mgl2* | 1.8 |
| *Mier2* | 1.5 |
| *Mmp14* | 1.9 |
| *Mmp2* | 1.5 |
| *Mmp23* | 1.7 |
| *Mmrn2* | 2.8 |
| *Mpp3* | 2.2 |
| *Mpped1* | 3.1 |
| *Mrgprf* | 1.8 |
| *Mrvi1* | 2.4 |
| *Msc* | 4.1 |
| *Mst1r* | 3.6 |
| *Msx1* | 2.9 |
| *Mtap7d1* | 1.5 |
| *Mtmr11* | 2.9 |
| *Mustn1* | 1.9 |
| *Myh10* | 1.8 |
| *Myh11* | 2.0 |
| *Myl9* | 1.5 |
| *Mylk* | 2.0 |
| *Myo7a* | 1.5 |
| *Nav1* | 1.9 |
| *Ncstn* | 1.5 |
| *Ndn* | 1.7 |
| *Ndrg1* | 1.6 |
| *Neo1* | 2.0 |
| *Neurl1a* | 2.3 |
| *Nfatc4* | 2.5 |
| *Nfix* | 2.2 |
| *Nkd1* | 3.7 |
| *Nkd2* | 1.6 |
| *Nkx2-3* | 2.2 |
| *Nlgn2* | 1.9 |
| *Nos3* | 1.7 |
| *Nos3* | 2.0 |
| *Nostrin* | 1.8 |
| *Notch4* | 2.9 |
| *Noxo1* | 2.0 |
| *Nr1d1* | 1.6 |
| *Nr1h3* | 1.8 |
| *Nr2f6* | 1.6 |
| *Nrarp* | 1.9 |
| *Nrbp2* | 2.4 |
| *Nxn* | 1.8 |
| *Oas2* | 3.2 |
| *Oaz2* | 1.6 |
| *Olfml2b* | 1.6 |
| *P2rx1* | 2.1 |
| *P2ry2* | 2.0 |
| *P4ha2* | 2.6 |
| *Pafah2* | 2.0 |
| *Palld* | 1.6 |
| *Palmd* | 1.8 |
| *Parm1* | 4.4 |
| *Parm1* | 7.9 |
| *Parva* | 2.1 |
| *Pcdh1* | 2.5 |
| *Pcdh1* | 3.1 |
| *Pdgfb* | 2.0 |
| *Pdlim3* | 2.2 |
| *Pdlim4* | 1.7 |
| *Pelo* | 1.8 |
| *Pfn2* | 1.7 |
| *Pgam2* | 1.9 |
| *Phlda3* | 1.8 |
| *Pi4k2a* | 1.5 |
| *Pigz* | 2.0 |
| *Pisd-ps1* | 4.7 |
| *Pkdcc* | 2.4 |
| *Pla2g2d* | 4.0 |
| *Plcd1* | 5.2 |
| *Pld1* | 1.9 |
| *Plec* | 1.6 |
| *Plekha4* | 2.2 |
| *Plekha6* | 1.8 |
| *Plekhh3* | 2.1 |
| *Plod3* | 1.5 |
| *Plvap* | 1.6 |
| *Plxna2* | 1.9 |
| *Pnck* | 2.3 |
| *Pnkd* | 5.7 |
| *Podn* | 1.7 |
| *Podxl* | 2.1 |
| *Ppap2b* | 1.6 |
| *Ppp1r14a* | 1.7 |
| *Ppp1r3c* | 1.5 |
| *Ppt2* | 1.6 |
| *Prg3* | 5.3 |
| *Prickle1* | 1.6 |
| ***Prnp*** | 1.5 |
| *Prosapip1* | 2.5 |
| *Prss23* | 2.4 |
| *Ptges3l* | 1.8 |
| *Ptgis* | 2.8 |
| *Pth1r* | 1.8 |
| *Ptms* | 1.6 |
| *Ptprm* | 1.6 |
| *Pvrl2* | 1.7 |
| *Rab11fip5* | 2.0 |
| *Rab3d* | 1.5 |
| *Rabl2* | 1.5 |
| *Rai14* | 1.8 |
| *Rap2a* | 2.0 |
| *Rapgef3* | 1.8 |
| *Rarres1* | 5.2 |
| *Rasd2* | 2.6 |
| *Rasip1* | 1.8 |
| *Rbfox1* | 7.0 |
| *Rbp1* | 2.0 |
| *Rbpms* | 2.0 |
| *Rem1* | 1.9 |
| *Renbp* | 1.6 |
| *Rep15* | 2.0 |
| *Rgl1* | 1.6 |
| *Rgma* | 2.0 |
| *Rgs9* | 2.2 |
| *Rhbdf1* | 1.5 |
| *Rhoj* | 1.7 |
| *Robo4* | 1.6 |
| *Rras* | 1.7 |
| *Rspo1* | 2.7 |
| *Rtn1* | 2.8 |
| *Rusc2* | 2.0 |
| *Ryr3* | 3.6 |
| *Scamp5* | 1.9 |
| *Scara3* | 1.9 |
| *Scarb1* | 1.7 |
| *Scarf2* | 2.1 |
| *Scd2* | 1.6 |
| *scl0004023.1_57* | 3.3 |
| *Sct* | 3.0 |
| *Sdc3* | 1.8 |
| *Sema3f* | 1.6 |
| *Sema5a* | 1.5 |
| *Sema6d* | 1.7 |
| *Serpina10* | 7.8 |
| *Serpinf1* | 2.1 |
| *Serpinh1* | 1.5 |
| *Sgk1* | 1.8 |
| *Shisa2* | 2.7 |
| *Siglec1* | 8.0 |
| *Slc16a9* | 1.7 |
| *Slc1a4* | 2.6 |
| *Slc22a17* | 3.3 |
| *Slc24a3* | 2.1 |
| *Slc24a6* | 1.7 |
| *Slc25a27* | 2.1 |
| *Slc2a1* | 1.6 |
| *Slc30a2* | 2.2 |
| *Slc4a3* | 2.2 |
| *Slc4a8* | 1.7 |
| *Slc7a4* | 1.8 |
| *Slc7a7* | 1.7 |
| *Slc9a3r2* | 1.6 |
| *Slc9a3r2* | 1.8 |
| *Slco2b1* | 1.7 |
| *Slitrk5* | 2.0 |
| *Smtn* | 2.1 |
| *Sncg* | 1.9 |
| *Snurf* | 1.6 |
| *Sod3* | 2.6 |
| *Sort1* | 1.5 |
| *Sox13* | 2.0 |
| *Sox17* | 2.2 |
| *Sox18* | 2.9 |
| *Sox5* | 1.7 |
| *Spon2* | 2.4 |
| *Spry4* | 2.0 |
| *Spsb1* | 1.7 |
| *Srgap3* | 2.1 |
| *Srr* | 1.6 |
| *St5* | 1.7 |
| *Stap2* | 1.9 |
| *Stard8* | 1.9 |
| *Stmn2* | 4.8 |
| *Stx1a* | 1.8 |
| *Stxbp1* | 1.6 |
| *Sulf1* | 2.8 |
| *Sv2b* | 2.8 |
| *Syde1* | 2.0 |
| *Synm* | 1.8 |
| *Syp* | 2.1 |
| *Tbx2* | 2.8 |
| *Tbx3* | 1.7 |
| *Tead2* | 1.9 |
| *Tek* | 1.9 |
| *Tesk1* | 2.2 |
| *Tgfb1i1* | 2.1 |
| *Tgfb1i1* | 2.2 |
| *Tgfb2* | 3.9 |
| *Tgfb3* | 1.9 |
| *Tgm1* | 2.2 |
| *Thsd1* | 1.6 |
| *Tie1* | 1.9 |
| *Timd4* | 5.3 |
| *Tle2* | 1.7 |
| *Tlr5* | 4.2 |
| *Tmem132a* | 1.7 |
| *Tmem132a* | 2.2 |
| *Tmem150a* | 2.6 |
| *Tmem184b* | 1.6 |
| *Tmem51* | 1.7 |
| *Tmem86a* | 1.9 |
| *Tnc* | 2.0 |
| *Tnfsf12-tnfsf13* | 1.9 |
| *Tnni3* | 1.8 |
| *Tnnt1* | 2.0 |
| *Tnnt2* | 2.8 |
| *Tpm1* | 1.7 |
| *Tpm2* | 2.0 |
| *Tppp3* | 2.0 |
| *Trim47* | 1.8 |
| *Trip10* | 1.6 |
| *Trp53inp2* | 1.8 |
| *Tspan10* | 3.8 |
| *Tspan15* | 2.1 |
| *Ttn* | 7.0 |
| *Uaca* | 2.2 |
| *Ube2e2* | 1.6 |
| *Uchl1* | 4.2 |
| *Unc13b* | 2.2 |
| *Unc5b* | 3.6 |
| *Unc5c* | 3.8 |
| *Upp1* | 2.2 |
| *Ushbp1* | 1.6 |
| *Vash2* | 3.3 |
| *Vasn* | 1.9 |
| ***Vegfa*** | 1.8 |
| *Vill* | 1.6 |
| *Vwf* | 1.6 |
| *Wbscr16* | 2.5 |
| *Wdr86* | 2.0 |
| *Wnt5a* | 1.9 |
| *Yap1* | 1.9 |
| *Zfp46* | 1.6 |
| *Zfp532* | 1.7 |
