# Supplementary material for: Clusterin Is a Potential Lymphotoxin Beta Receptor Target That Is Upregulated and Accumulates in Germinal Centers of Mouse Spleen during Immune Response
Source: PLoS One. 2014 May 27;9(5):e98349. doi: 10.1371/journal.pone.0098349 (PMC4035297; doi:10.1371/journal.pone.0098349)
Supplement: Table S2 — The list of genes which mRNA levels were more than 1.5-fold lower in wild type spleen stroma comparing to LTβR-KO spleen stroma. (DOCX) [file pone.0098349.s003.docx]

**Table S2**

The list of genes which mRNA levels were more than 1.5-fold lower in wild type spleen stroma comparing to LTβR-KO spleen stroma.

| Gene name | WT:LTβR-KO ratio |
| --- | --- |
| *1810009J06Rik* | 3.9 |
| *Agtr1a* | 1.5 |
| *Ccl11* | 6.4 |
| *Ces1d* | 4.1 |
| *Cfd* | 1.8 |
| *Clcn3* | 1.7 |
| *Clcn3* | 1.9 |
| *Col3a1* | 1.7 |
| *Cyp2s1* | 1.6 |
| *Ddx26* | 1.8 |
| *Dpt* | 1.8 |
| *Fcrl1* | 4.2 |
| *Kel* | 1.9 |
| *Ccl8* | 4.0 |
| *Igkv4-72* | 5.6 |
| *Mat2a* | 2.0 |
| *Mmp3* | 2.5 |
| *Mpo* | 1.6 |
| *Pnliprp2* | 3.6 |
| *Cxcl7* | 1.6 |
| *Pvalb* | 7.5 |
| *Sit1* | 3.3 |
| *Slc16a6* | 1.8 |
| *Spink3* | 6.8 |
| *Sult1a1* | 1.6 |
